# Exposure to aflatoxin B_1_ and associated risk factors in hepatitis C patients in cosmopolitan city of Pakistan: facility-based study

**DOI:** 10.11604/pamj.2021.40.247.23396

**Published:** 2021-12-21

**Authors:** Muhammad Ayaz Mustufa, Zubia Zia, Rabia Ilyas, Rehan Khan, Syed Naim Ul Hasan Naqvi, Firdous Imran Ali

**Affiliations:** 1 Baqai Institute of Pharmaceutical Sciences (BIPS), Baqai Medical University, Karachi-74600, Pakistan,; 2 Health Research Institute, National Institute of Health, Islamabd-46000, Pakistan,; 3 Department of Bio-Chemistry, University of Karachi, Karachi-75270, Pakistan,; 4 The Directorate of Anti-Quackery, Sindh Healthcare Commission, Head Office, Karachi-1293, Pakistan,; 5 Department of Chemistry, University of Karachi, Karachi-75270, Pakistan

**Keywords:** Aflatoxin B1, hepatotoxic, hepatocarcinogenic, hepatitis C-infected patients, risk factor

## Abstract

**Introduction:**

population-based follow-up study has been designed to investigate the contributing factors to high exposure to Aflatoxin B_1_ (AFB_1_) and the subsequent associated risk factors among hepatitis C-infected patients at a referral centre, Karachi. Pakistan. Hepatitis C infection affects millions of individuals worldwide and confers high morbidity and mortality, especially in lower middle-income countries (LMICs) including Pakistan. A literature review of recent studies has revealed that a number of hepatocellular carcinomas (HCC) cases are markedly increased in Pakistan, where one of the potential causes of HCC is hepatitis C virus. The objectives of this study were to determine frequency of Aflatoxin B_1_ (AFB_1_) exposure and other associated characteristics among hepatitis C patients at a referral centre, Karachi, Pakistan.

**Methods:**

a semi-structured pre-coded pro forma designed to collect socio-demographic, Pharmacological, biochemical and clinical information from patients and hospital records. Patient´s pre and post polymerase chain reaction (PCR), serum alanine aminotransferase (ALT) levels and other blood parameters were analysed. AFB_1_ exposure was determined using an ELISA kit and validated through high-performance liquid chromatography (HPLC).

**Results:**

AFB_1_ exposure was found in 30 (34%) patients. Post treatment responders were 49 (55.6%). More than 37% of study participants had a family history of hepatitis C. About 74% had a history of surgical procedure, and around 36% of study participants had a blood transfusion history. Up to 36% participants were fond of spicy food and around 25% study participants were eating roadside food on daily basis.

**Conclusion:**

high frequency of AFB_1_ exposure due to risky dietary habits, low level of formal education and awareness are contributing factors may be responsible for high exposure of AFB_1_. Effective and multidimensional strategies are needed to prevent advance stage progression of disease and associated complications.

## Introduction

Hepatitis C virus (HCV) infection affects more than 175 million people worldwide. HCV is one of the major contributing factors of liver damage along with the development of hepatocellular carcinoma (HCC) [1]. Studies showed that the number of hepatocellular carcinoma cases is markedly increased in Pakistan, where one of the potential causes of HCC is hepatitis C virus [2]. In most of the cases, hepatitis C is a progressive disease and various factors are responsible for its progression, including co-existence of other disease such as hepatitis B, human immunodeficiency virus (HIV), schistosomiasis, etc. Lifestyle modifications such as alcohol abuse, insulin resistance, smoking, consumption of food contaminated with various toxins specifically AFB_1_ also contributing to disease progression widely [3, 4]. Combination therapy of pegylated interferon alpha with ribavirin have shown significant improvement in therapeutic outcomes of the disease, showing between 44 to 69% sustained Virological Response rate (SVR) [5-8]. Recently, addition of direct acting antivirals (DAAs) protease inhibitors to the standard treatment shown improved SVR in patients with HCV genotype1 and 3a, further studies are going on to evaluate the long-term effect of these drugs [9-11]. Aflatoxin (AF) in food is one of the serious threats that are posed to human health on the global level, particularly aiming the countries that are under development Liver serves as the place where the metabolism of AF takes place [12].

AF is the potent carcinogenic agents that mainly damage the liver as the chronic exposure of AF with high levels are possessed with adequate association with growth alteration in adolescent along with immune suppression [13]. AFB_1_ that has been reported for carcinogenic activity, playing a vivacious role in the hepatic disease progression [14, 15]. Substantial exposure of AFB_1_ to HCV positive patient may lead to progressive liver disease or cirrhosis or even development of HCC [16, 17]. In Pakistan, AFB_1_ contaminated food is a serious threat. High levels of this toxin have been reported in various food commodities, especially in Chilies, which are to exceeding allowable limits by European Union [18]. The metabolites of AFB_1_ have the ability to react with DNA guanine residues, this is associated with mutation of TP53 gene at codon 249 accompanying exposure to AF and HCC [19]. Such modulation of DNA activity by AFB_1_ has a strong role in inducing carcinogenesis [20, 21]. It is estimated that about 85% of the cases of HCC are reported in the countries with lower income as these countries are at the constant mainstay of exposure to risk factors posed by dietary AFB_1_ and viral infections including chronic hepatitis B and C [22].

## Methods

**Setting:** it was a cross-sectional study carried out at PMRC research centre, NICH Karachi, Pakistan and Department of Chemistry, University of Karachi for period of 24 months.

**Sample size:** sample size was considered on the basis of National survey of hepatitis B and C, i.e., 4.2% prevalence of HCV in Karachi. Sample size was calculated at 95% confidence interval with 4% precision, using EPI software 6. Sample size calculated as 97= 100 and 10% for non-compliance. Therefore, the final sample size for the current study is 110.

**Sampling technique:** the purposive sampling technique was used, hepatitis C patients visiting gastroenterology and hepatology centre at public facility for treatment will be included as per inclusion and exclusion criteria.

**Blood sampling:** following standard protocol, a phlebotomist collected 3 to 5 ml blood in an air sterile container to avoid any contamination.

**Blood samples collection procedure:** personal hygiene and cleanness of the blood collecting area was ensured to avoid blood contamination. Disposable gloves and hand sensation lotion were used to maintain sterility of the procedure. Before collecting the blood the skin was cleaned properly with an alcohol swab, as per need double cleaning was also done with another alcohol swab to ensure complete disinfection of the skin. A tourniquet was used to make the vein prominent, the needle is gently inserted in the popped up vein the tourniquet is released, and the blood is gently drawn in to the blood collection tube. Care was taken to collect the blood in the first attempt to avoid double puncturing of the veins. The blood was transferred gently into the blood collection tube.

**Blood samples processing:** the blood collection tube was labelled with a cryo makers, the tube was left undisturbed on the work bench, after which it was centrifuged. The centrifuge was set at 3000 rpm for 10 minutes to get clear serum. Blood sample were further subjected for biochemical analysis including Serum ALT and Aflatoxin B_1_ levels and rest was used for *qPCR*. All consumables used for blood collection and separation, essentially discarded in puncture resistant danger bins. Care was taken not to touch the red cells with the tip of the dropper to avoid breakage of red cells.

**Inclusion criteria:** hepatitis C positive patients with a positive PCR report for HCV ribonucleic acid (RNA); patients who were not under treatment for last 6 months to avoid interference of drug that may give false results and patients using WHO recommended treatment were included. The same time, stage of disease was confirmed from attending physician on clinical basis. Deviation in treatment protocol was considered as drop out from the study.

**Exclusion criteria:** patients who refuse to participate; patients suffering from any significant congenital anomaly/life-threatening disease and patients who do not had PCR based confirmed hepatitis C positive results.

**Study sites:** the study was conducted in collaboration with Department of Chemistry, University of Karachi, Baqai Institute of Pharmaceutical Sciences and PHRC, Research Centers situated at Jinnah postgraduate medical college (JPMC) and National Institute of Child health (NICH), Karachi, Pakistan. Ethical approval has been taken from the institutional ethical review board (IERB) of the National Institute of Child Health (NICH) and all methods and protocols were performed in accordance with the relevant standard guidelines and regulations. After explanation of the underlying purpose of the research to the participants, informed written consent was obtained. All the participants were above 18 years of age and consent to participate in the study. In order to avoid chances of data breaching, coding was done for the maintenance of confidentiality of the participants.

**Patients selection and pre-treatment analysis:** during the initial screening, 108 patients having positive HCV antibody test were selected and further tested for HCV RNA test based on criteria set by the centers for disease control and prevention (2013). Out of 108 Patients, 96 were found positive for HCV RNA test, enrolled for the study. An HCV RNA test was carried out through PCR using QIA amp DSP Virus Spin Kit by Qiagen. Pre-coded pro forma were used for collection of data, including demographic, behavioural, clinical and biological data. Patients were advised to reach within the hour at the respective facility after eating a routine meal for serum AFB_1_ determination. Pre-treatment serum ALT levels and HCV RNA were also recorded.

**Aflatoxin B_1_ analysis:** AFB_1_ was assessed with the help of Elisa kit of the Bio Scientific Corporation (MaxSignal® Aflatoxin B_1_ ELISA Test Kit Manual - 1055-01). The Proportion of positive samples were confirmed by HPLC with fluorescence detector qualitatively [23, 24].

**Post treatment analysis:** after 6 months, 88 patients followed the prescribed regimen for the treatment appropriately, while the others were not able to continue the treatment and follow up mainly because either they move back to their home town (interior Sindh). Finally, the patient´s blood samples, i.e. 5 ml taken for post treatment analysis of HCV RNA and serum ALT levels to observe the treatment outcomes of HCV positive patients and results were computed.

**Procedure/methods in details:** this was a cross-sectional analytical study, completed in 24 months. Cross-sectional studies are useful in providing an overall estimate of prevalence, exposure, outcome and coverage in a given context. A structured questionnaire was used to record socio-demographic and possible risky behaviours including dietary habits, nature of work, socio-cultural issues, etc. of study participants. Considering the availability of patient pool; study subject selection and biological sample (blood) collection in sterile containers were done at PMRC research centre. qPCR analysis was done under the supervision of Co-PI. Chemical analysis of all samples was performed at University of Karachi under the direct supervision of PI.

Permission to conduct the study was taken from the head of participating Institutions. Potential study participants, visiting at Gastroenterology and Hepatology centre in public facility, were contacted to explain the motive of the study and invited to participate. Structured informed written consent was obtained from each study individual. A pre-coded questionnaire was filled for those who consented to participate. Phlebotomist drew the blood for following investigations; pre *qPCR* analysis to confirm the viral load. That was conducted at PMRC Research Laboratory, under supervision of PI/CO-PI as per convenience; serum ALT were determined using *Microlab-300* at PMRC Research Centre, Karachi; aflatoxin B_1_ by using *gradient HPLC* at Department of Chemistry, University of Karachi and after sample collection, patients were provided standard treatment as per guideline by attending physician. After treatment, the patient´s blood sample was collected again to validate treatment outcome by viral load using *qPCR*.

**Ethical review:** to comply with the ethical principle of beneficence; the study subjects were compensated by performing mandatory *qPCR* test free of cost. Informed written consent was obtained from potential participants after explaining the purpose of the study before inclusion. The participants had the right to disassociate from the study at any time; confidentiality was maintained by coding and ethical clearance was taken from IERC to conduct the study (IERB No. 15/2014).

### Statistical analysis

**Data management plan:** the following protocol was adopted for study participants to finalize the results.

**Data processing:** coding of filled pro forma to maintain the confidentiality was done to maintain the confidentiality. A log book was used for daily record keeping.

**Editing, coding and data transferring:** all data collected was cross-checked by field supervisors on a daily basis and weekly transferred to the data management cell from the participating stations. Prior to data entry, all forms were checked for completeness and consistency as well as coding of open-ended responses and area codes, etc. In case of inconsistency or missing responses, the editors flagged the errors/omissions and consulted the interviewers for possible explanations.

**Data analysis:** tabular presentation of data IBM SPSS statistics SPSS 20 was used for data processing and chi-square test was applied to acceptable p-value ≤ 0.05 for a possible association between different parameters.

**Ethics approval and consent to participate:** clearance was obtained from the Institutional Ethical Research Board (IERB) of the host Institute (IERB No. 15/2014). Informed written consent was obtained from each individual for participation. Research involving human participants and/or animals: Not applicable.

## Results

Socio-demographic details of study participants and category wise AFB_1_ exposure are presented in Table 1. More than 35% of the study participants were in between 40-49 years of age. Whereas, only 3.4% of patients were under 20 years of age. The majority of participants (54.5%) were not formally educated. The income level of the study participants reveals that 47.7% of them were earning, salary up to 10,000 Pakistani rupees (100 USD). More than half (55.7%) patient lives in proper house facilities while 39.8% lived in an un-plastered houses and the rest 4.5% lived in semi plastered houses.

**Table 1:** physiognomies of patients and AFB_1_ exposure

Physiognomies of Subjects	N	Percentage %	Exposure to AFB_1_ (%)	No Exposure to AFB_1_ (%)
**Age**				
18-19	3	3.4	-	3(100)
20-29	12	13.6	3(25)	9(75)
30-39	18	20.5	7(38)	11(62)
40-49	31	35.2	12(39)	19(61)
50-59	18	20.5	8(44)	10(56)
> 60	6	6.8	-	6(100)
**Gender**				
Male	36	40.9	11(31)	25(69)
Female	52	59.1	19(37)	33(63)
**Educational Standing**				
None	48	54.5	17(35)	31(65)
Can read &write	5	5.7	1(20)	4(80)
Primary	11	12.5	4(36)	7(64)
< Matric	6	6.8	1(17)	5(83)
Matric	6	6.8	3(50)	3(50)
Under graduate	6	6.8	0	6(100)
Graduate	5	5.7	4(80)	1(20)
Post graduate	1	1.1	0	1(100)
**Income of Individual**				
<10000	42	47.7	17(40)	25(60)
11000-20000	22	25.0	4(18)	18(82)
21000-30000	15	17.0	7(47)	8(53)
>30000	9	10.2	2(22)	7(78)
**House Type**				
Kacha	35	39.8	10(29)	25(71)
Pacca	49	55.7	18(37)	31(63)
Kacha & Pacca	4	4.5	2(50)	2(50)
**Religion**				
Islam	84	95.5	30(36)	54(64)
Chrisianity	4	4.5	0	4(100)
**Mother Tongue**				
Sindhi	10	11.4	1(10)	9(90)
Urdu	22	25.0	10(45)	12(55)
Punjabi	16	18.2	5(31)	11(69)
Saraiki	4	4.5	3(75)	1(25)
Pushto	17	19.3	5(29)	12(71)
Balochi	8	9.1	3(38)	5(62)
Hindko	10	11.4	3(30)	7(70)
Others	1	1.1	0	1(100)

The majority of the study population, i.e. 95%, belongs to the religion Islam and 25% of patients were Urdu speakers. A total of 30 HCV patients were found to be exposed to (≥ 5 ng/ml) levels of AFB_1_. Post treatment PCR results revealed that 55.7% (49) of HCV patients responded to the given treatment against HCV whereas, 44.3% (39) didn't respond well. Pre-treatment serum ALT levels of the enrolled patients showed that 33% (29) had normal serum ALT levels and 67% (59) patient had raised levels of serum ALT. Post treatment serum ALT levels reflect normal in 62.5% (55) patients. While, 37.5% (33) patients had high ALT levels (Table 2).

**Table 2:** clinical and biochemical presentation

Parameters	Responders (49)		Non-Responders (39)		
	Normal	Abnormal	Normal	Abnormal	p-value
Pre-Hemoglobin	28(57%)	21(43%)	20 (51.3%)	19(48.7%)	0.58
Post-Hemoglobin	17(34.6%)	32(65.4%)	16(41%)	23(59%)	0.54
Bilirubin	00	49(100%)	00	39(100%)	0.91
Conjugated Bilirubin	(92%)	(8%)	30(77%)	9(23%)	0.05*
ALT	17 (34.7%)	32 (69.3%)	12 (30.8%)	27 (69.2%)	0.69
Alkaline Phosphatase	13 (26.5%)	36 (73.5%)	10 (26%)	29 (74%)	0.93
GGT	37 (75.5%)	12 (24.5%)	33(84.6%)	6(15.4%)	0.29
Albumin	47(96%)	2(4%)	37(95%)	2 (5%)	0.82
Globulin	47(96%)	2(4%)	39(100%)	--	0.20
Total Protein	49(100%)	--	39(100%)	--	0.91
Serum Creatinine	36(73.5%)	13(26.5%)	24(61.5)	15(38.5%)	0.23
Uric Acid	33(67%)	16(33%)	39(100%)	--	0.001*
Pre- WBC´s	40(81.6%)	9(18.4%)	29(74%)	10(26%)	0.41
Post- WBC´s	40(81.6%)	9(18.4%)	31(79.5%)	8(20.5)	0.80
Pre- Platelets	38(77.5%)	11(22.5%)	33(84.6%))	6(15.4%)	0.40
Post- Platelets	36(73.4%)	13(26.6%)	30(77%)	9(23%)	0.07
HBA1c	25(51%)	24(49%)	13 (33.3%)	26(66.6%)	.05*
Prothrombin time	35(71.4%)	14(28.6%)	10(26%)	29(74%)	.001*

**Figure 1:**
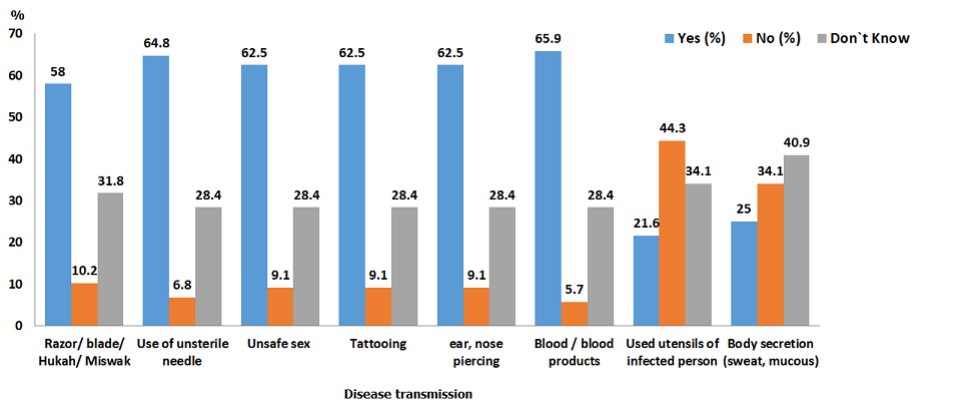
knowledge assessment of hepatitis C patients regarding disease transmission

**Figure 2:**
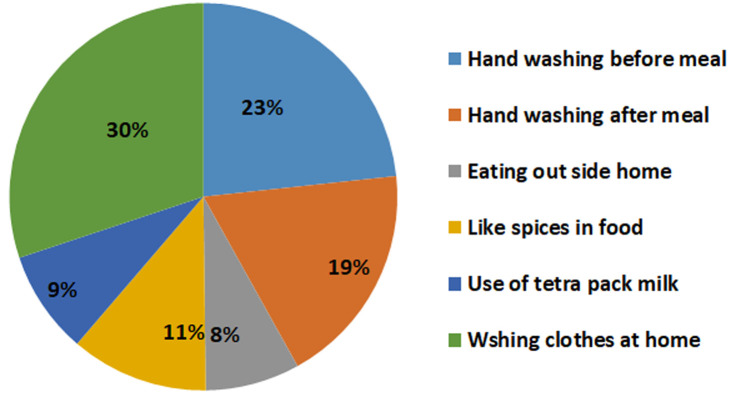
life style patterns of hepatitis C patients

**Figure 3:**
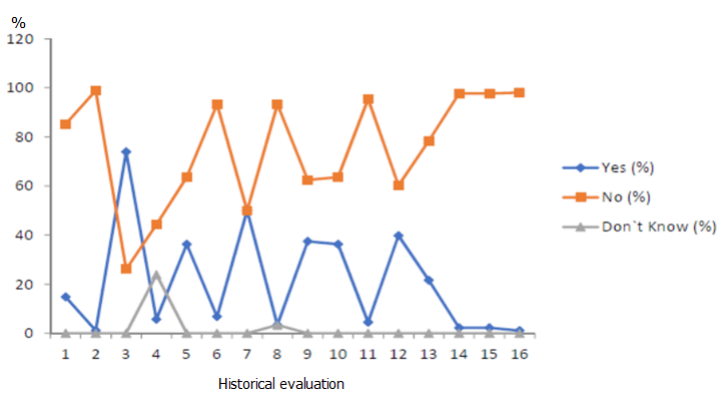
historical evaluation of hepatitis C patients with information about other hepatitis C infected members in family and other risks

Various factors responsible for the transmission of hepatitis C virus and awareness level among the enrolled patients were also assessed and represented in Figure 1, Figure 2, Figure 3. Patients found partially aware of the fact that the disease could be transmitted through the reuse of infected implements such as razor, blade, miswak (i.e. 58%), the use of unsterile syringes i.e. 64.8%, unsafe sex, i.e. 62.5%, reuse of instruments utilized in the process of tattooing (62.55), ear, nose piercing (62.5), inappropriate blood transfusion process 65.9%. Many of the patients do believe that disease can also be spread through sharing of infected person's utensils 21.6%, as well as body secretion including sweat, mucous i.e. 25%.

## Discussion

Pakistan sharing very high burden of hepatitis C patients and progression of liver disease up to a higher stage (hepatocellular carcinoma) in a short duration is a focal concern. Therefore, following work was aimed to adopt a multidimensional approach, to know the role of contributing factors responsible for this disease synergy [25]. Our study revealed a high frequency of AFB_1_ exposure among hepatitis C positive patients with comparatively low compliance to treatment. The close correlation of AFB_1_ with progression of liver cancer is well known, and intake of AFB_1_ through dietary routine items is also very well documented in many parts of the world [26-28]. Due to lack of knowledge and consumption of contaminated food items, AFB_1_ remains a major cause of disease outbreaks and progression.

Similarly, notable levels of AFB_1_ (33%) and very low level of education with more than 60% of patients had no records of formal education are considerably, leading concerns in our study population, may be responsible for high AFB_1_ exposure and hence leading to progression of disease. Therefore, it is the ultimate need to draw attention to public sensitization and strategies to reduce AFB_1_ production from dietary items to ensure the safety and quality of food. Treatment response cannot be generalized only to AFB_1_ exposure; there are a number of other factors, including age, type of genotype, duration and stage of disease, environmental effects, genotype specific pharmacological targets, clinical findings, Co infection of HIV, diabetes, smoking, etc. [29-33]. Pretreatment Virological testing was done for confirmation of all anti HCV positive patients, and treatment response was categorized as a responder and non-responders on the basis of viral load after completing course. Other biochemical and clinical parameters were also used to categorize the chronicity of disease for each individual.

As mentioned in Table 2, there was no obvious difference observed among treatment responders and non-responders, as reported earlier. While, significant difference between prothrombin time (P < .001), uric acid (P < .001), and patients with raised Hba1C levels (P < .05) showed low treatment adherence among non-responders [32, 33]. More than 60% cases of our study above 40 years of age, suggesting declining trends in younger age groups; but still a proportion of cases remain high and requires more efforts to reduce it to negligible number. Very low level of formal education with only around 6% passed the 14 standards of formal education among study population, indicating the low compliance of massive awareness schemes may be due to generalizability, unconsciousness and perceived mastery at each level of dissemination. The majority of our participants (90%) belonged to the lower socio-economic strata with remuneration of less than 300 USD per month and more than half of these families earning only up to 100 USD per month, implying that, poverty is more prevalent and can be considered as a contributing factor.

**Factor responsible for disease progression:** working on geographic ancestry, it was alarming to observe that more than 90% of Sindhi-speaking patient had AFB_1_ exposure; may be due to poor hygiene, contaminated use of food and more prevalent roadside eating habits. A group of workers proposed a solution to curtail AF contamination in wheat using solar and blue light, can be considered as one of the low cost alternates through public sensitization [34, 35]. Awareness and risk assessment of HCV infected patients highlighted the high exposure of risk factors responsible for transmission of hepatitis C virus, including; needle stick injury (21.6%), receipt of potentially contaminated blood through surgical/dental procedures (73.9%), etc. As highlighted in Figure 2, low adherence to handwashing, eating spicy/junk food outside the home, etc. essentially requires lifestyle modifications to adopt a harm reduction strategy among concerned community and the public. Limitations of this study include; we did not perform biopsies/fibro scan of patients to correlate clinical staging and disease chronicity. Similarly, results may not be generalized due to the hospital setting and may not be comparable with reference to AFB_1_ exposure, dietary habits, etc. to other groups due to no control arm. Molecular analysis for confirmation of progression of disease was not done.

## Conclusion

Finally, it is imperative to reduce the future burden of HCV related morbidity and mortality through contextually focused harm reduction strategies for disease management, including associated risks of AFB_1_ exposure, poor hygiene, risky dietary habits and treatment of such patients must not be limited to ARTs only. Simultaneously, lifestyle modification through hygienic and nutritious diet plans may be adopted by contextually focused community-based awareness strategies at mass level to avoid disease progression and improve prevailing management outcome of treatment.

### What is known about this topic

- *AFB_1_-induced hepatocarcinogenesis among hepatitis C-infected patients has not been extensively studied yet;*
- *AFB_1_ exposure has been one of the potential causes of hepatic diseases in Pakistan due to low level of food safety knowledge in food handlers and poor hand hygiene*.

### What this study adds

- *This cohort study can aid in improving clinical management in hospitals for slowing disease progression and to reduce complications by minimizing these exposures*.
